# Non-Bactericidal Antifouling Coating Inspired by the “Swinging Effect” of Coral Tentacles in Waves

**DOI:** 10.3390/biomimetics10090606

**Published:** 2025-09-10

**Authors:** Yue Yin, Jianfu Wang, Xu Zheng

**Affiliations:** 1 School of Civil Engineering, Jilin Jianzhu University, Changchun 130118, China; zhengxu_2005@126.com; 2 Key Laboratory of Bionic Engineering, Ministry of Education, Jilin University, Changchun 130022, China; wjianfu.0105@outlook.com

**Keywords:** coral tentacles, bionic, environmental protection, “swinging effect” antifouling

## Abstract

Inspired by the free swing of coral tentacles driven by water currents to actively repel microbial attachment, we have identified a unique physical anti-fouling strategy: coral “swinging effect” anti-fouling. Taking the fleshy soft coral (*Sarcophyton trocheliophorum*) as an example, its surface is covered with numerous soft tentacles. These coral tentacles utilize the force of water current fluctuations to freely sway, resembling a “feather duster” waving to repel microorganisms attempting to settle and establish themselves. Based on this characteristic, this study delves into the living habits of corals, observing the expansion and contraction cycles of their tentacles. Simultaneously, simulations of the anti-fouling performance of coral tentacles were conducted. It demonstrates that the “swinging effect” of the tentacles can effectively prevent the attachment of fouling organisms. Furthermore, this study uses *S. trocheliophorum* as a biomimetic prototype to design and prepare an artificial coral-mimic substrate (ACMS). It employs the common marine Gram-negative bacterium *Paracoccus pantotrophus* as a microbial sample to test anti-fouling performance in both pure static water environments and low-flow water environments. The results showed that the 13 mm-long ACMS could bend and overlap the surface of the rear tentacles to the greatest extent under the unidirectional scouring action of low-speed water flow (3.5 m/s), forming an anti-fouling protective layer. Additionally, the “swinging effect” phenomenon generated by the tentacles under water flow scouring demonstrated excellent anti-fouling effects. This study not only provides further evidence for research on coral antifouling performance but also offers new concepts and ideas for antifouling strategies in low-flow water environments, such as stationary ships in ports and underwater infrastructure facilities at docks.

## 1. Introduction

Marine microorganisms attach themselves to the surface of underwater substrates, gradually forming large areas of biofouling, which poses a serious threat to ships, port infrastructure and the marine ecological environment. For example: (i) Fouling organisms attach to the bottom of ship hulls, increasing the ship’s weight and resistance to navigation, while accelerating fuel consumption. This not only results in multiple emissions of harmful gases (NO_x_, CO_2_, SO_x_) but also causes significant economic losses [1,2,3,4]. (ii) Fouling organisms secrete mucus to tightly adhere to the hull or underwater equipment surfaces. The components of the mucus accelerate corrosion of ships and pipeline casings, significantly shortening the service life of equipment [5,6]. (iii) Fouling organisms attached to the ship’s hull can enter different marine areas as the vessel navigates. Due to the absence of natural predators, new species can proliferate extensively, leading to biological invasions and disrupting marine ecosystems [7,8]. Therefore, combating biological fouling has become an indispensable issue in safeguarding marine environments and protecting human interests.

In the early days, people typically added toxic components such as copper, mercury, and tributyltin to anti-fouling coatings to reduce the attachment of fouling organisms [9,10,11,12]. However, with the increasing strictness of environmental protection laws and regulations regarding the use of toxic anti-fouling agents, as well as the enhanced resistance of fouling organisms to anti-fouling agents, single-toxic anti-fouling methods have gradually been phased out [13,14,15,16]. In recent years, to align with environmental policies, researchers have achieved some success through anti-fouling methods such as modifying the surface of anti-fouling coatings [17], constructing micro/nano-scale surface structures [18], and establishing electrostatic interactions in coatings [19]. However, in low-flow water environments or when vessels are stationary, the absence of water flow shear force makes it difficult for existing antifouling coatings to actively repel fouling organisms. Therefore, there is an urgent need to develop antifouling strategies for vessels operating at low speeds or in stationary states, as well as for underwater fixed equipment and facilities at ports and terminals.

**Scheme 1:**
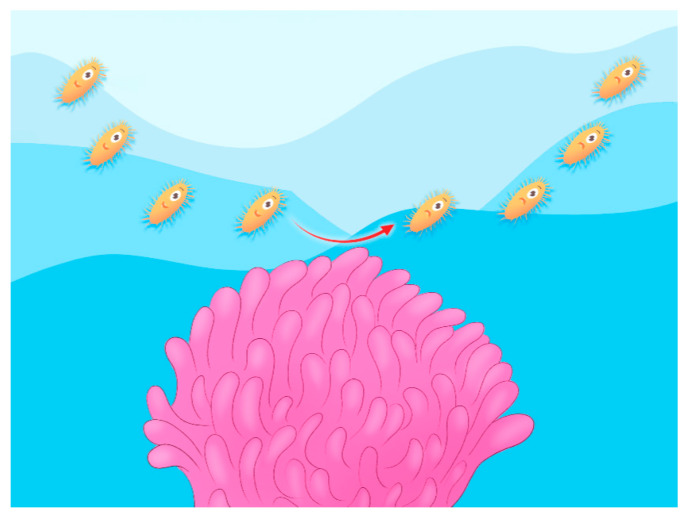
Mechanism of “swinging effect” antifouling of coral tentacles in marine environment.

This paper focuses on coral, a common marine organism. Coral grows permanently on reefs in tropical waters. Unlike marine organisms such as sharks and dolphins, which can reduce fouling by swimming rapidly and using water currents to scrub away fouling organisms, coral surfaces remain clean and free of fouling organisms [6,15,20,21,22,23,24]. Research has found that certain coral species release mucus from their surfaces to form a self-enveloping state when exposed to external environmental stimuli or unstable marine conditions. Once the environment stabilizes, the outer mucus layer peels off, and a new coral epidermis forms. This process can be viewed as a form of physical anti-fouling achieved through molting and “changing clothes” [25,26]. Some coral species can also emit a faint glow, creating a “fluorescent effect” that effectively inhibits the approach of diatoms and other planktonic organisms [27,28]. In addition to the anti-fouling characteristics of the above two types of coral, we observed that the surface of *S. trocheliophorum* coral is covered with long and dense tentacles. The tentacles freely sway with the waves, actively driving away microorganisms attempting to settle. We refer to this as the “swinging effect” anti-fouling strategy (Scheme 1).

In order to investigate the “swinging effect” of coral tentacles, we conducted ecological observation experiments on the extension patterns and morphological characteristics of coral tentacles. We found that coral tentacles are normally extended, with each tentacle exhibiting a conical shape that is thinner at the top and thicker at the bottom. The length of the tentacles is mostly between 7 mm and 13 mm. The tentacles are randomly arranged with close spacing. To visually verify the anti-fouling performance of the coral tentacles’ “swinging effect”, we used a fine sand suspension to simulate the anti-fouling performance of coral tentacles against marine microorganisms. The experimental results showed that the tentacles freely swayed under the influence of water flow fluctuations, forming a sweeping pattern that significantly reduced the deposition of fine sand on the inner surface of the coral. This demonstrates that the tentacles serve as an important component of the coral’s self-anti-fouling mechanism.

Based on the aforementioned research findings, this study employs biomimetic similarity principles and prior experimental results from the research team to select room-temperature vulcanizing silicone rubber (RTV-2) containing 0.36%wt graphene as the matrix material. Through template fabrication, ACMSs with different tentacle structures (tentacle length, spacing) are prepared. RTV-2 containing 0.36%wt graphene has a lower surface free energy and elastic modulus, which can help the material actively release attached fouling organisms [22,29,30]. Additionally, this material can undergo greater elastic deformation under the influence of water flow fluctuations, better simulating the swaying state of coral tentacles in waves.

This study selected the common marine Gram-negative bacterium *P. pantotrophus* to test the anti-fouling performance of ACMSs. In dynamic tests, ACMSs demonstrated fewer bacterial deposits than samples without tentacles through the “swinging effect”. Among these, ACMSs with tentacles 13 mm long and spaced 2 mm apart exhibited the best anti-fouling performance. Analysis indicates that the “swinging effect” is most pronounced in longer tentacles. Additionally, under unidirectional water flow, the longer and denser tentacle layers can overlap and cover the surfaces of subsequent layers, forming an anti-fouling protective layer. This dual protection maximizes anti-fouling effectiveness. Compared to traditional anti-fouling coatings with added biocides and modern physical eco-friendly anti-fouling coatings, ACMSs utilizing the tentacle “swinging effect” can actively repel the attachment of fouling organisms in low-flow water environments, providing a new approach for anti-fouling methods for underwater fixed equipment and facilities, and holding significant engineering application value.

## 2. Materials and Methods

### 2.1. Coral Rearing Environment and Test Equipment

#### 2.1.1. Coral Rearing Environment

**Figure 1:**
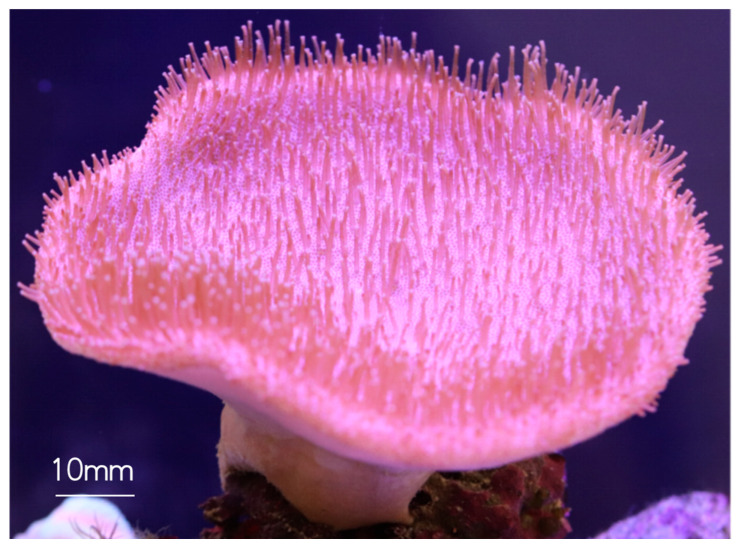
Photograph of the soft coral *S. trocheliophorum*.

In this experiment, we selected the fleshy soft coral (*S. trocheliophorum*), which has long tentacle structures (Unit: millimeters), as the research subject (Figure 1). We designed and constructed an aquaculture tank with a post-filter system in the laboratory, which can simulate a marine environment suitable for coral growth. (Figure S1, Supporting Information). This system automatically removes impurities from the water and facilitates biological degradation, ensuring the stability of the artificial seawater ecosystem. Based on the growth habits and living environment of the coral, the temperature of the aquaculture tank was maintained at 25 °C, and the salinity was controlled between 1.02 and 1.03 g/cm^3^.

#### 2.1.2. Coral Biological Observation Test Equipment

The wave-making system was purchased from Zhongke Ecology’s SLIM 6000 device (Shenzhen, China), with a maximum power of 9 W, a minimum power of 3 W, and a flow output of 6000 L/h. It has four modes: wave-making, transmission, flow-making, and turbulence. In working condition, the power output of the wave-making system can be adjusted to 40%, 60%, 80%, and 100%, respectively.

The coral-specific LED light ZA1201 was purchased from Guangzhou Jack Lighting Electronics Co., Ltd. (Guangzhou, China). It can simulate five natural ecological light modes: “dawn”, “sunrise”, “daytime”, “sunset” and “nighttime” (Table S1, Supporting Information). Through changes in lighting, the rhythmic expansion and contraction of coral tentacles in a natural environment could be observed. However, during subsequent photography, different light modes may result in varying colors in the coral photographs.

### 2.2. Simulation of Natural Environment—Coral Tentacles Expansion and Contraction Regularity Test

Under conditions that minimize external disturbances to the coral’s living environment, LED lights were used to simulate five different lighting modes suitable for coral survival: “dawn”, “sunrise”, “daytime”, “sunset” and “nighttime”. Using the time-lapse photography function of a digital camera, coral samples were photographed every 10 min. Then, record and analyze the morphological changes in the coral tentacles and crown over a 24 h period.

### 2.3. Fluid Medium Disturbance Environment—Coral Tentacles Expansion and Contraction Regularity Test

Coral tentacles are normally extended in the “daytime” lighting mode of LED lights. At this time, the initial state of coral tentacles was photographed and recorded. Subsequently, the distance between the fixed wave-making system and the coral is about 30 cm. Turn on the wave-making system, select the wave-making mode, and set the output power to 40%, 60%, 80%, and 100%, respectively. The experiment used a digital camera to record the expansion and contraction of coral tentacles under fluid medium disturbances at different power levels, as well as the recovery of coral tentacles after the wave-making motion stopped.

### 2.4. Coral Tentacles “Swinging Effect” Antifouling Performance Simulation Test

To verify whether the “swinging effect” produced by coral tentacles under water flow fluctuations can prevent microbial attachment. This paper uses yellow micro-sand particles to simulate microorganisms in seawater and tests the anti-fouling performance of the “swinging effect” of the coral tentacles. (i) Grind yellow micro-sand into tiny particles using a grinding bowl and filter through a 50-mesh fine sieve. The sand particles are approximately 0.28 mm in diameter. (ii) Weigh 200 milliliters of artificial seawater and 50 g of ground yellow fine sand in a test cup, and thoroughly mix them to form a fine sand suspension. (iii) This test compares the anti-fouling performance under both extended and contracted tentacle states. Set the wave-making system output power to 60%, and use a syringe to draw 10 milliliters of fine sand suspension, then spray it 10 cm above the coral. Subsequently, take photographs and conduct comparative analysis.

### 2.5. Design and Preparation of ACMSs with the “Swinging Effect” Based on Coral Tentacles

#### 2.5.1. Materials

RTV-2 and curing agent (ethyl orthosilicate) were purchased from Jiacheng Electronic Materials Co. (Shenzhen, China). Acetone and anhydrous ethanol were purchased from Beijing Chemical Group Co. (Beijing, China).The silane coupling agent (KH-550) was purchased from Nanjing Genesis Chemical Auxiliaries Co. (Nanjing, China).Tetrahydrofuran was purchased from Shanghai Maclean Biochemical Technology Co. (Shanghai, China). Bacterial strain ATCC 35,512 (*P. pantotrophus*) was purchased from Chuanxiang Biotechnology, Ltd. (Shanghai, China). All aqueous solutions were prepared using ultrapure water (18.2 M Ω, Milli-Q, Millipore). All chemical reagents were used directly without further purification.

#### 2.5.2. Design and Processing of the ACMSs

Based on the results of coral biological observation experiments, this paper sets the tentacle lengths of ACMSs to 7 mm, 10 mm, and 13 mm, respectively, with tentacle spacings of 2 mm, 2.5 mm, and 3 mm, respectively, and arranged in an orderly manner. The tentacle morphology was optimized to have a base diameter of 1 mm and a tip diameter of 0.8 mm, forming a biomimetic coral tentacle structure resembling a cone. Based on the above data, nine molds with different tentacle size mirror structures were constructed, and ACMSs were prepared using the template method. (Figure S2, Supporting Information). The ACMSs synthesized using these molds can be characterized for surface morphology using a digital camera. (Figure S3, Supporting Information). The preparation process is the same as in our previous work [22]. To facilitate mold processing and removal, white resin material was selected for 3D printing to prepare the molds in this experiment.

### 2.6. Bacteria Culture and the ACMSs Antifouling Performance Test

#### 2.6.1. Bacterial Culture

Single colonies of *P. pantotrophus* on solid ATCC 1396 medium agar plates were transferred to 20 mL liquid medium and grown at 30 °C for 24 h under 200 rpm rotation. Then the bacteria were diluted to 10^6^ cfu mL^−1^ with broth. The culture protocol was the same as we reported [22,28].

#### 2.6.2. Antifouling Performance Test

In this study, *P. pantotrophus*, a common Gram-negative bacterium found in the ocean was selected as the microbial agent for the antifouling test. The antifouling performance of ACMSs was tested in both static water environments and simulated low-flow marine water environments. Graphene composite films (GCFs) without tentacle structures were selected as the control group. To facilitate the distinction between test samples, they were numbered according to Table 1.

In the static water environment test, all test samples were immersed in the *P. pantotrophus* bacterial solution environment for 96 h. To reduce test errors, we prepared three parallel samples for each specification. Remove the soaked test samples and gently rinse their surfaces with phosphate-buffered saline (PBS) to remove any excess bacterial solution floating on the sample surface. Then, place the samples in sterile centrifuge tubes and add 4 milliliters of PBS buffer solution. Use the ultrasonic cleaner to subject the centrifuge tube to 30 min of ultrasonic agitation, causing bacteria adhering to the sample surface to be transferred into the PBS buffer solution via ultrasonic agitation. Finally, determine the bacterial count remaining on the sample surface by measuring the optical density at a wavelength of 600 nanometers (OD_600_).

In dynamic water environment testing, a dynamic anti-fouling rotating scouring device was used to simulate low-speed ocean currents. This device uses rotational motion instead of linear motion to simulate the scouring effect of unidirectional water flow on ACMSs during low-speed ship travel. (Figures S4 and S5, Supporting Information). In this experiment, three samples of each type were prepared to reduce experimental error. The test samples were evenly arranged and fixed on the test carrier plate of the rotating scouring device, and the rotation speed was set to 3.5 m/s. Test samples were obtained on the 3rd, 6th, and 9th days, which were tested using the same method as the static test. The number of bacteria on the sample surface was determined by measuring the optical density OD_600_, and digital photographs were taken for comparison and analysis.

## 3. Results and Discussion

### 3.1. Natural Environment Simulation—Coral Tentacle Expansion and Contraction Patterns & Biological Sample Information Acquisition

**Figure 2:**
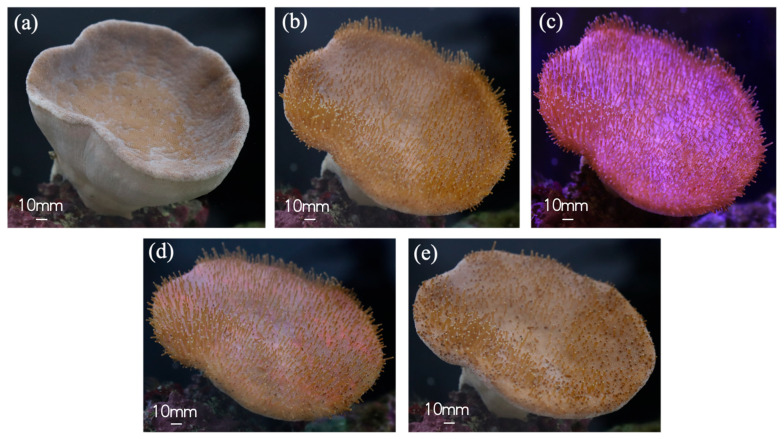
(**a**–**e**). Schematic diagrams of the coral changes under “dawn”, “sunrise”, “daytime”, “sunset”, and “nighttime” lighting period.

Observation shows that the expansion and contraction of coral tentacles is closely related to time changes. During the “dawn” light period, the coral forms an arch-shaped structure, with the head crown contracting inward, all tentacles retracting, and dense granular traces of tentacle contraction remaining on the outer surface. (Figure 2a). During the “sunrise” period, the coral head crown and tentacles gradually expand from the inside out, but remain in a retracted state. (Figure 2b). During the “daytime” period, the coral head crown and tentacles are fully extended, and the densely growing tentacles sway continuously under the influence of water currents. (Figure 2c). As time passes, during the “sunset” period, the coral’s head crown remains in an outwardly extended state. However, some tentacles have entered a dormant phase, and the dense layer of tentacles gradually becomes sparse. (Figure 2d). Until the “nighttime” lighting period, the coral head crown gradually closes inward, and the coral surface also shows uneven distribution of short tentacles and patchy marks due to the large-area retraction of the tentacles. (Figure 2e).

Analysis of the daily habits of corals over a 24 h period shows that during the “sunrise”, “daytime”, and “sunset” periods, the tentacles and head crowns of corals remain extended. The recording time was approximately 20.5 h. During the “dawn” and “nighttime” periods, the contraction process of the coral head crown and tentacles lasted only 3.5 h. Therefore, we can say that the extension of the tentacles is the normal state of coral under normal living conditions.

Based on the above observations, corals as marine organisms that grow fixed to reefs, cannot swim away to avoid the attachment of fouling organisms. We boldly hypothesize that, under normal conditions, the dense growth of coral tentacles on the surface may form a dynamic layer through oceanic fluctuations, which could actively repel microorganisms attempting to attach and establish themselves. Of course, coral’s anti-fouling mechanisms cannot be determined solely by the movement of tentacles. For example, our previous research has explored topics such as coral mucus antibacterial properties and the anti-adhesive properties of coral’s elastic surface layer [31]. However, the concept of using the “swinging effect” formed by coral’s long tentacle structure to actively repel fouling organisms has significant implications for anti-fouling strategies for underwater structures at port terminals and slow-moving vessels.

**Figure 3:**
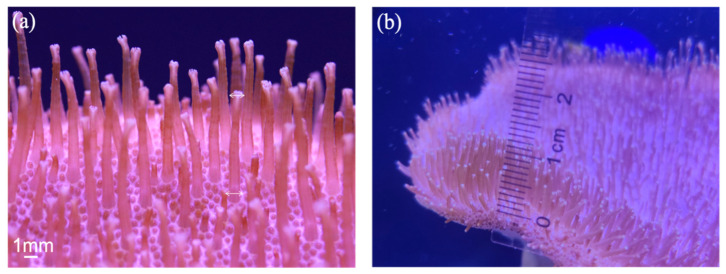
(**a**) Structural proportions of the coral tentacles; (**b**) Length measurements of the coral tentacles.

This paper obtains ecological information about corals under normal states (tentacles fully extended). At this time, the tentacles appear as conical shapes that are thinner at the top and thicker at the bottom. Through pixel estimation analysis using Photoshop CC 2019 software, it was found that the diameter of the base of the coral tentacles is approximately 1 mm, and the diameter at the tip is approximately 0.8 mm. (Figure 3a). Due to the refractive effects of the coral aquaculture tank and seawater, visual estimation of coral tentacle length can result in significant errors. Therefore, a ruler was placed in the water for comparison with the photographed images. The results showed that the majority of tentacle lengths were concentrated between 7 mm and 13 mm, with the tentacles exhibiting a random distribution pattern and close spacing. (Figure 3b). Obtaining ecological information about coral tentacles provides a theoretical basis for the subsequent development of ACMSs.

### 3.2. Fluid Medium Disturbance Environment—Coral Tentacle Response Regularity

**Figure 4:**
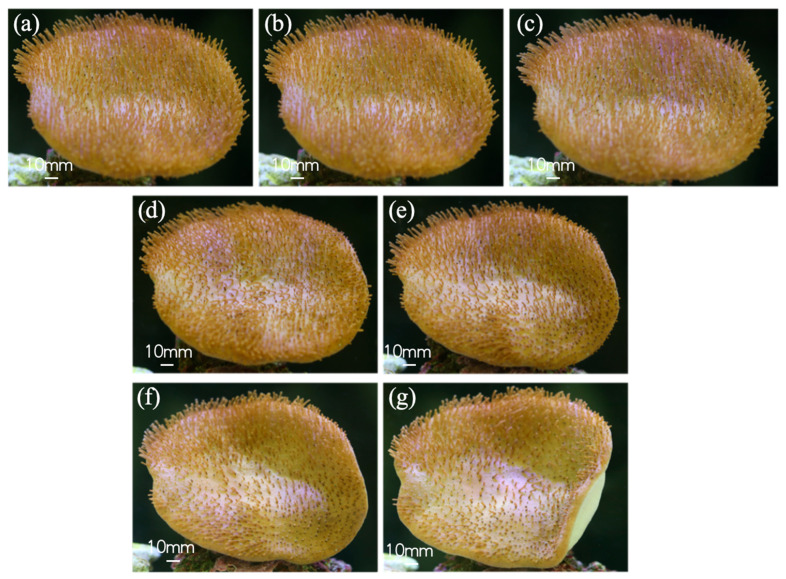
(**a**) Normal tentacle extension state; (**b**) Tentacle changes at 40% wave-making power output; (**c**) Tentacle changes at 60% wave-making power output; (**d**) Tentacle changes at 80% wave-making power output; (**e**) Stable state of the tentacle at 80% wave-making power output; (**f**) Tentacle changes at 100% wave-making power output; (**g**) Stable state of the tentacle at 100% wave-making power output.

Preliminary experiments have shown that coral tentacles can fully extend under normal conditions and produce a “swinging effect” in response to waves. However, the impact of wave intensity on the coral itself requires further investigation. This study selected the “daytime” period when the tentacles are fully extended (Figure 4a), and observed changes in tentacle morphology by controlling the output power of the wave-making system. (i) For a wave-making power output of 40%, the coral tentacles swayed with the waves, showing no significant contraction changes within 5 min (Figure 4b). (ii) When the output power was increased to 60%, the swaying amplitude of the coral tentacles increased and they tilted toward the wave flow direction, yet the tentacles still showed no significant contraction changes within 5 min (Figure 4c). This indicates that coral tentacles can sustainably function to protect the coral surface under general ocean current impacts. (iii) Upon further increasing the wave-making system output power to 80%, the coral exhibited self-protective characteristics under strong current impacts, with partial tentacles rapidly contracting within 10–30 s, and localized patchiness appearing at the central surface of the coral crown (Figure 4d). After maintaining wave intensity for 5 min, the length and density of the tentacles no longer undergo significant changes, indicating that the coral is in a stable state (Figure 4e). Although the coral retracts some tentacles under strong current impacts due to its self-protective mechanism, the relatively intact tentacle protective layer remains intact and continues to perform its function of removing microorganisms. (iv) When the output power was increased to 100%, the phenomenon of tentacle retraction in the middle of the coral surface became more obvious under extreme wave impact (Figure 4f). After 5 min of continuous wave impact, the phenomenon of tentacle retraction gradually stabilized, and the head crown tended to contract inward (Figure 4g).

**Figure 5:**
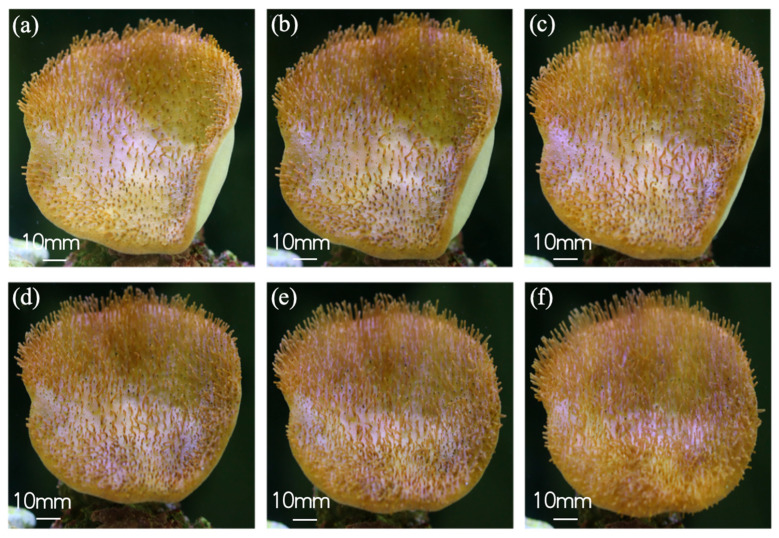
(**a**) Initial state of coral after cessation of water flow fluctuations; (**b**) Coral recovery state after 20 min; (**c**) Coral recovery state after 30 min; (**d**) Coral recovery state after 40 min; (**e**) Coral recovery state after 50 min; (**f**) Coral recovery state after 60 min.

After ceasing the water flow fluctuations from the wave-making system, the recovery of coral tentacles and head crowns was documented via digital camera every 10 min. Initially, the entire coral remained in a self-protective state with inward retraction (Figure 5a). After 20 min, the coral persisted in this self-protective state with no significant changes (Figure 5b). After 30 min, the coral’s head crown gradually extended outward, though tentacle extension remained subtle (Figure 5c). By 40 min, the head crown had fully transitioned from concave to outward extension. Short tentacles began emerging from mottled areas on the coral surface (Figure 5d). After 50 min, the density of tentacles on the coral surface increased, with continuous extension occurring. (Figure 5e). After 60 min, both the density and length of the tentacles had returned to normal levels. (Figure 5f).

The aforementioned experiments demonstrated that by altering the intensity of fluid medium fluctuations, observations of coral tentacle morphological changes revealed that under general and stronger fluctuation conditions, coral tentacles did not exhibit widespread retraction. Furthermore, the tentacles could sway with the wave currents, providing effective protection for the coral’s surface. Under extreme wave conditions, although coral triggers its self-protection mechanism to retract part of its tentacles, after the wave stimulation stops, the tentacles can return to their normal state within a short period of time. Based on this, it not only reaffirms that the extension of coral tentacles is the normal state but also suggests that the “swinging effect” of tentacles plays a crucial role in coral’s anti-fouling mechanism.

### 3.3. Coral Tentacles “Swinging Effect” Antifouling Performance Test

The marine ecological environment is extremely complex. In addition to the large marine organisms we are familiar with, such as dolphins, whales, and sharks, there are also parasitic organisms that attach themselves to these creatures—fouling organisms. During their initial formation, fouling organisms exist in the form of bacteria and planktonic spores, seeking stable surfaces and secreting chemical mucus to gradually expand their habitat. Dynamic surfaces, however, can effectively prevent this [19,28,29]. This study uses a sand particle suspension to simulate marine microorganisms and verifies the anti-fouling performance of the dynamic “swinging effect” formed by coral tentacles through wave action.

**Figure 6:**
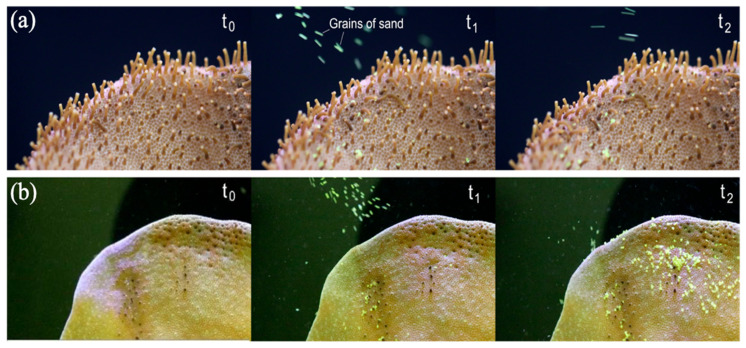
(**a**,**b**) Sand particle adhesion with/without the “swinging effect” of tentacles.

Under normal conditions, the coral tentacles extend, and the waves generated by the wave-making system cause the fine sand suspension to approach the coral, allowing observation of sand particle attachment (Figure 6a). As shown in the figure, at the initial time t_0_, when no fine sand suspension is sprayed, the coral continues to sway under the influence of the wave current. At t_1_, the fine sand suspension is carried toward the coral by the wave currents, and the coral uses the “swinging effect” of its tentacles to repel the yellow sand particles. At t_2_, only a small number of sand particles settle on the inner surface of the coral.

In the same manner, we conducted comparative experiments during the coral dormant period (tentacle retraction) and observed the attachment of sand particles (Figure 6b). At t_0_, when no fine sand suspension was sprayed, the coral surface was very clean. At t_1_, yellow sand particles were freely carried toward the coral by wave currents. At this point, without the “swinging effect” of tentacles, yellow sand particles continued to accumulate on the coral surface. By t_2_, when the spraying ended, a significant amount of yellow sand particles had already settled on the coral surface.

The test results show that the “swinging effect” produced by coral tentacles under the action of waves can effectively repel fine sand particles that attempt to settle. By analogy, it can also repel microorganisms that attempt to attach themselves, forming a good anti-fouling protective layer.

### 3.4. Anti-Fouling Performance Test of the ACMSs

The ACMSs were designed and prepared based on the theory of biomimetic similarity. Static and dynamic water environment tests were conducted separately.

**Figure 7:**
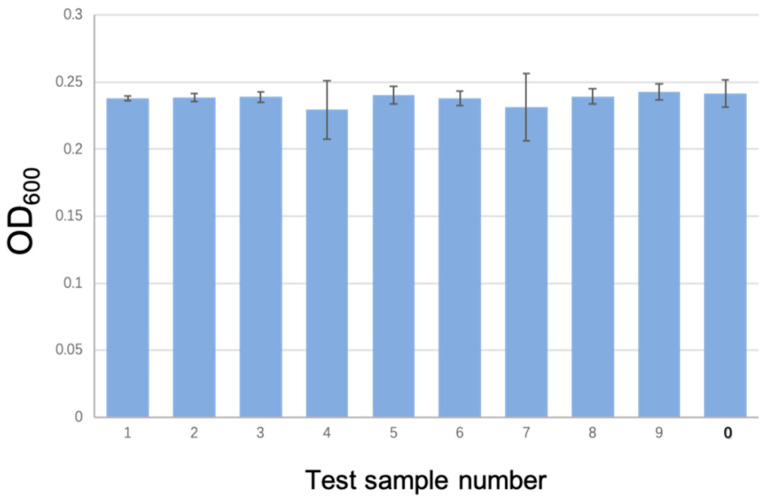
Test results of OD_600_ values of bacterial adherence in static tests under *P. pantotrophus* bacterial solution environment.

Static test results in the *P. pantotrophus* bacterial environment showed that there was no significant difference in the number of bacteria attached to the ACMSs compared to GCF. There were also no significant differences between ACMSs with different tentacle parameters. In optical density (OD_600_) measurements, all values were between 0.2 and 0.25 (Figure 7). This indicates that in a static water environment, tentacle structures without the “swinging effect” cannot effectively demonstrate anti-fouling performance.

**Figure 8:**
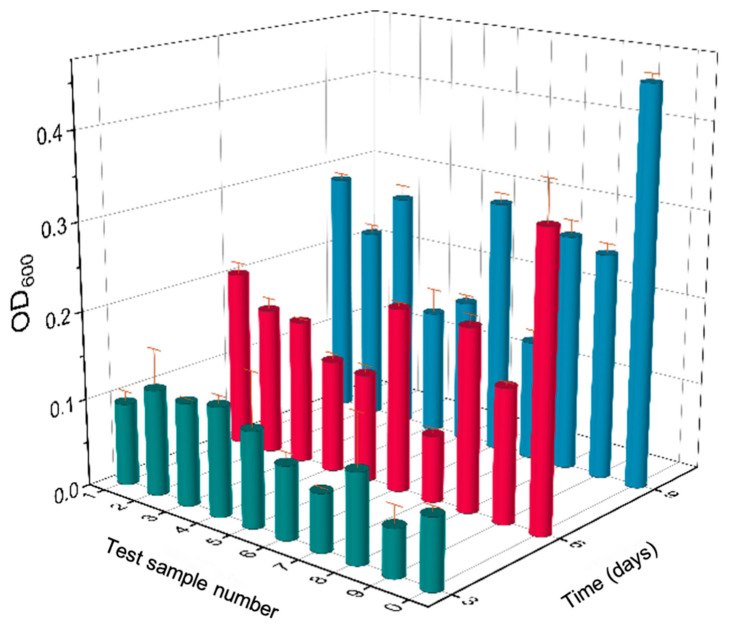
Test results of OD_600_ values of bacterial adherence in dynamic tests under *P. pantotrophus* bacterial solution environment.

In the dynamic anti-fouling performance test, the same *P. pantotrophus* bacterial environment was selected, and the anti-fouling performance was determined by measuring the number of bacteria attached to the surface of the test sample using optical density (OD_600_). The test results were plotted as a 3D histogram (Figure 8).

The results showed that on the 3rd day, the bacterial adhesion values of all test samples were around 0.1, with only slight fluctuations. Based on subsequent image comparison analysis and speculation, it is difficult for bacteria to adhere stably over a large area in a short period of time. Furthermore, due to differences in tentacle structure, when the bacterial adhesion value is low, judging anti-fouling performance solely based on bacterial adhesion value is prone to error.

On the 6th day of the experiment, the OD value of GCF (No. 0) without tentacle structures was significantly higher than 0.3, far exceeding that of ACMSs with tentacle structures. This indicates that the “swinging effect” brought about by tentacle structures has already demonstrated good anti-fouling performance in the short to medium term. Meanwhile, the differences in anti-fouling effects among the ACMSs with different tentacle parameters have become increasingly apparent. For example, the OD values of experimental groups 1, 6, and 8 were significantly higher than those of other groups during the same period, and all three groups had tentacle lengths of 7 mm. This may be due to the insufficient “swinging effect” of short tentacles under the action of water flow.

On the 9th day of the experiment, the OD value of the GCF without tentacle structures (No. 0) had already approached 0.5, once again exceeding the bacterial adhesion levels on the surfaces of all the ACMS samples during the same period. This indicates that, in a dynamic aquatic environment, the “swinging effect” induced by the tentacles of the ACMSs have shown preliminary effectiveness in achieving long-term anti-fouling properties. Further analysis of anti-fouling performance under different tentacle parameters revealed that the 7th experimental group (tentacle length 13 mm, spacing 2 mm) had the lowest bacterial adhesion and the best anti-fouling effect. This indicates that dense, long tentacles can effectively prevent bacterial settlement under unidirectional water flow erosion, forming a stable anti-fouling protective layer.

**Figure 9:**
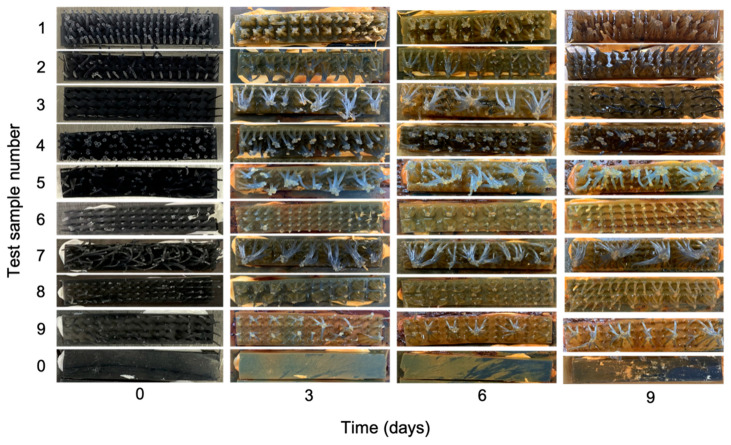
Comparison of dynamic antifouling test samples in *P. pantotrophus* bacterial solution environment.

In addition to the OD value measurement method, we used a digital camera to synchronously record the bacterial adhesion status at each experimental time point and conducted a visual analysis (Figure 9).

At 3 days, the GCF of the 0th experimental group showed a more pronounced yellow bacterial film covering the surface compared to other ACMSs. In the 6th and 9th experimental groups, due to the wider tentacle spacing, the basal surfaces also exhibited varying degrees of bacterial coverage at the tentacle spacing locations. Although the tentacle spacing in the 1st experimental group was relatively dense, the shorter tentacle length may have prevented the full realization of the “swinging effect,” resulting in more bacterial adhesion on the tentacle tips. The 5th and 7th experimental groups had longer tentacle structures with denser tentacle spacing. Consequently, no significant bacterial adhesion was observed on either the tentacles or the substrate base.

On the 6th day, the experimental group 0 surface accelerated bacterial deposition and had already turned brownish-yellow. Surfaces of experimental groups 1, 6, and 8 also showed varying degrees of yellow bacterial attachment, with all three groups having tentacle lengths of 7 mm. In contrast, experimental groups 3, 5, and 7, which had tentacle lengths of 13 mm, remained largely clean. This indicates that within a short timeframe, the “swinging effect” of longer tentacles can effectively resist stable bacterial attachment, forming an effective self-cleaning mechanism.

On the 9th day, the surface of experiment group 0 had turned dark brown, indicating that the bacterial adhesion had reached a high value and formed a biofilm, which meant that the anti-fouling performance of the GCF had basically failed. Compared with the GCF and other experiment groups, the tentacles and substrate of group 7 remained relatively clean. This group was the ACMS with a spacing of 2 mm and tentacle length of 13 mm.

**Figure 10:**
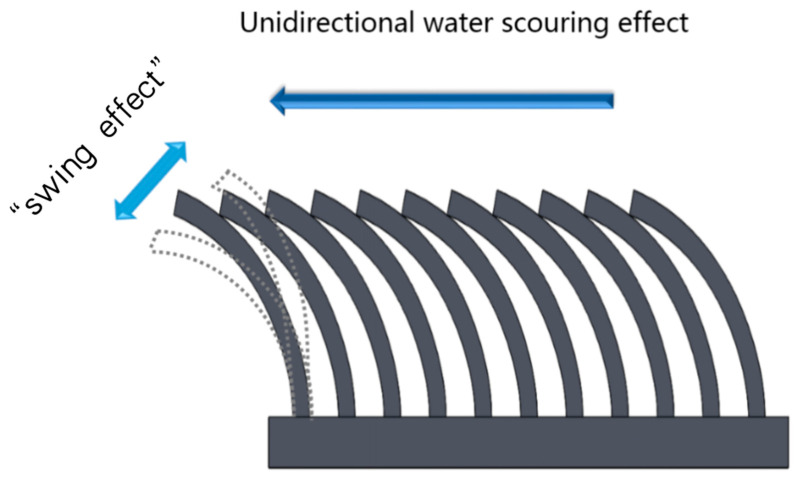
Schematic diagram of the ACMS tentacle’s “overlay protection” and “swinging effect”.

Observations revealed that the 13 mm-long tentacle structures can bend to their maximum extent and cover the rear tentacle surface under the unidirectional erosion of water flow, forming an anti-fouling protective layer. Additionally, the tentacles can effectively generate a “swinging effect” under water flow fluctuations to deter bacterial attachment. Reducing the spacing between tentacles minimizes gaps on the ACMS surface, thereby maximizing the anti-fouling protective function of the tentacles. (Figure 10).

## 4. Conclusions

This study observed the free swinging motion of coral tentacles, resembling the movement of a feather duster, as they actively repel microorganisms attempting to attach themselves, thereby forming an effective anti-fouling mechanism. We refer to this phenomenon as the “swing effect.” Using *S. trocheliophorum* as the biological sample, we simulated real oceanic conditions with specialized coral rearing equipment to observe the morphology and dimensions of tentacles under normal conditions. It was found that tentacle lengths predominantly ranged between 7 and 13 mm, densely covering the coral surface. Individual tentacles exhibit a tapered conical shape, narrower at the tip (approximately 0.8 mm diameter) and wider at the base (approximately 1 mm diameter). Furthermore, under varying wave intensities, the coral tentacles maintained normal extension and oscillation. Only after exposure to extreme wave stimulation (“wave-making” output power at 100%) did a brief self-protective retraction occur, followed by the ability to extend the tentacles again. Through these experiments, we gained deeper insights into coral behavior, confirming tentacles as a vital component. To visually demonstrate the relationship between tentacle swinging and anti-fouling, this study used a yellow sand suspension to simulate microorganisms. Results validated that the “swinging effect” of real coral tentacles effectively prevents the settling of these “microorganisms”. 

Based on the principle of biomimetic similarity, ACMSs were prepared in this study. Anti-fouling performance tests were conducted using the common marine Gram-negative bacterium *P. pantotrophus* in both static and dynamic aquatic environments. Comparing dynamic and static tests reveals that in purely static water environments, bacterial adhesion on ACMSs showed no significant difference from GCF, with OD_600_ values concentrated between 0.230 and 0.245. In contrast, during dynamic testing, the tentacle structures of ACMSs generated a “swinging effect” through water flow fluctuations. Compared to concurrent GCF test samples, surface bacterial deposition on ACMSs was significantly reduced. Notably, ACMSs with tentacles measuring 13 mm in length and 2 mm spacing exhibited the lowest bacterial attachment (OD_600_ value of 0.1), demonstrating exceptional antifouling performance. Analysis of the experimental conclusions indicates that the 13 mm-long, 2 mm-spaced ACMS tentacles (the group with optimal anti-fouling performance) maximize the “swinging effect”. Furthermore, under unidirectional water flow, these tentacles exhibit directional bending, partially overlapping adjacent tentacles. Specifically, the tips of upper-layer tentacles cover the bases of lower-layer tentacles and the substrate surface. This overlapping configuration directly prevents physical contact between bacteria and the substrate surface. Combined with the “swinging effect”, it creates dual physical anti-fouling protection.

In summary, drawing inspiration from the free oscillation of coral tentacles in nature, this paper employs natural principles to prepare ACMS that generate an anti-fouling “swinging effect”. This mechanism effectively prevents the stable attachment of marine microorganisms and bacteria, establishing an environmentally friendly physical anti-fouling system. This novel concept offers a promising approach for anti-fouling methods in ships operating at low speeds and underwater equipment at port terminals, demonstrating significant engineering application value.

## Figures and Tables

**Table 1:** Test sample number. Unit: mm.

	Parameter	Tentacle Distance	Tentacle Length
Number			
1		2	7
2		2.5	10
3		3	13
4		2	10
5		2.5	13
6		3	7
7		2	13
8		2.5	7
9		3	10
0		0	0

## Data Availability

The original contributions presented in this study are included in the article/Supplementary Materials. Further inquiries can be directed to the corresponding author.

## Supplementary Materials

The following supporting information can be downloaded at: https://www.mdpi.com/article/10.3390/biomimetics10090606/s1, Figure S1: Schematic diagram of the overall structure of the back-filtered coral rearing tank; Figure S2: 3D printed mirror molds of the ACMSs; Figure S3: Schematic diagram of the ACMSs sample; Figure S4: Schematic diagram of the dynamic antifouling rotating scouring device; Figure S5: Physical view of dynamic antifouling rotating scouring device; Table S1: Coral special LED light 5 kinds of light mode corresponding time.
