# Supplementary material for: Non-Bactericidal Antifouling Coating Inspired by the “Swinging Effect” of Coral Tentacles in Waves
Source: Biomimetics (Basel). 2025 Sep 10;10(9):606. doi: 10.3390/biomimetics10090606 (PMC12467940; doi:10.3390/biomimetics10090606)
Supplement: Supplementary file 1 [file biomimetics-10-00606-s001.zip › biomimetics-3843456-supplementary.pdf]

Supporting Information

Non-bactericidal antifouling coating inspired by the “swinging effect” of coral tentacles in waves

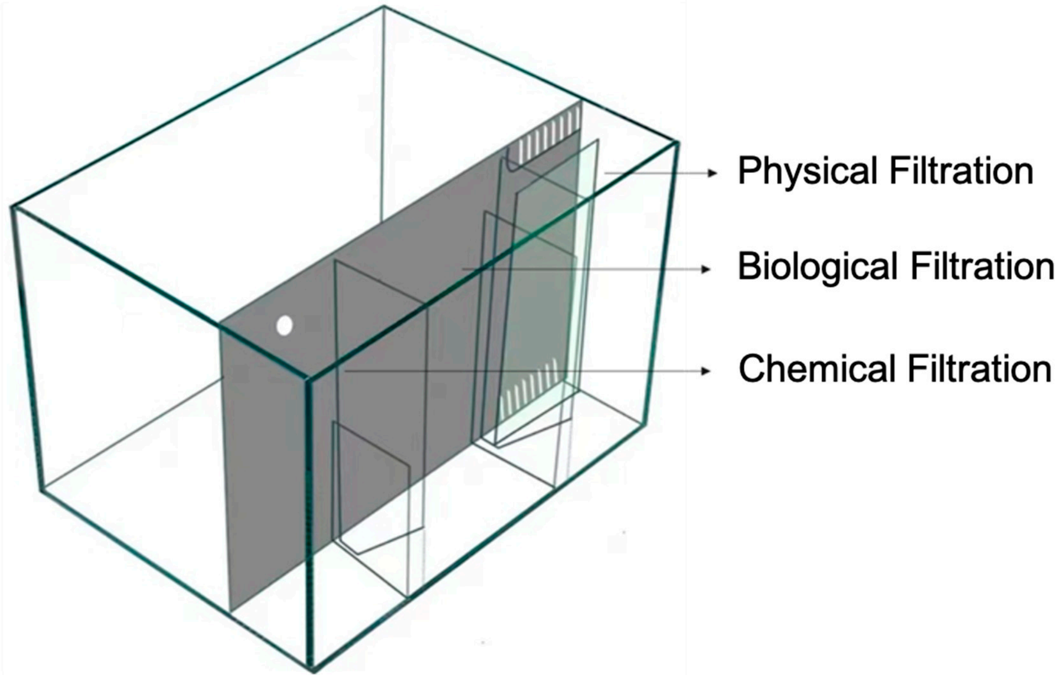

Figure S1. Schematic diagram of the overall structure of the back-filtered coral rearing tank.

① Biological filtration includes: nitrification system, denitrification system and plant adsorption system. Its oxidation of organic matter by aerobic bacteria converts the toxic nitrite oxygen into non-toxic nitrate. The anaerobic denitrifying bacteria then reduce the nitrate to nitrogen and release it back into the recirculation system. ② Physical filtration includes: live rock, filter cotton and protein separator. It can filter solid impurities in the water tank and play a role in ensuring clear water and translucent water quality. ③ Chemical filtration includes: phosphoric acid filter. It can play the role of biodegradation and stabilize the ecological balance of seawater.

Table S1.

Table S1 Coral special LED light 5 kinds of light mode corresponding time.

Light mode	Time period
Wawn	00:00-03:00
Sunrise	03:00-08:00
Daytime	08:00-14:00
Sunset	14:00-18:30
Nighttime	18:30-24:00

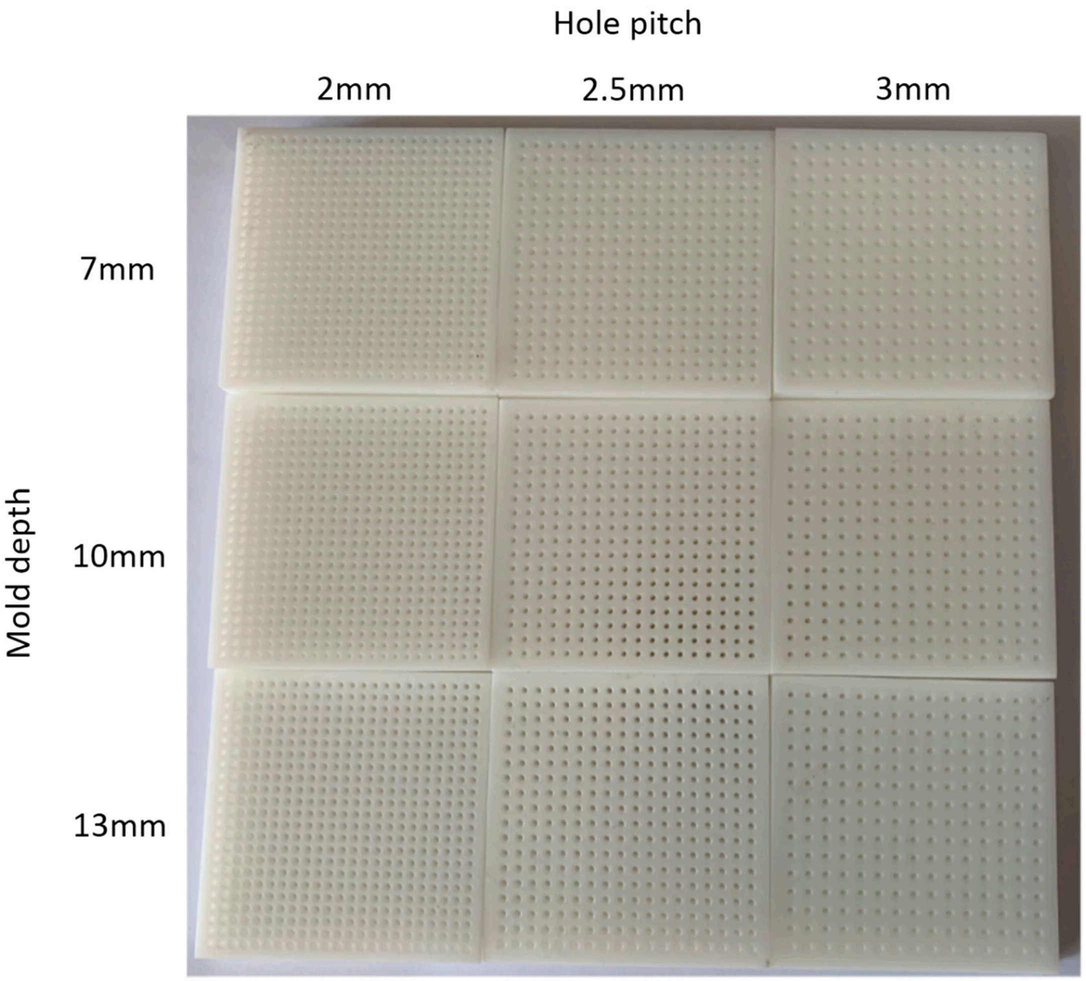

Figure S2. 3D printed mirror molds of the ACMSs.

According to the structural design of the ACMSs, the mirror mold tentacle lengths were determined to be 7 mm, 10 mm, and 13 mm; the spacing were 2 mm, 2.5 mm, and

3 mm, and the overall structure was through-hole. The bottom diameter of the tentacles were 1 mm and the top diameter were 0.8 m. The length and width of the molds were both 50 mm.

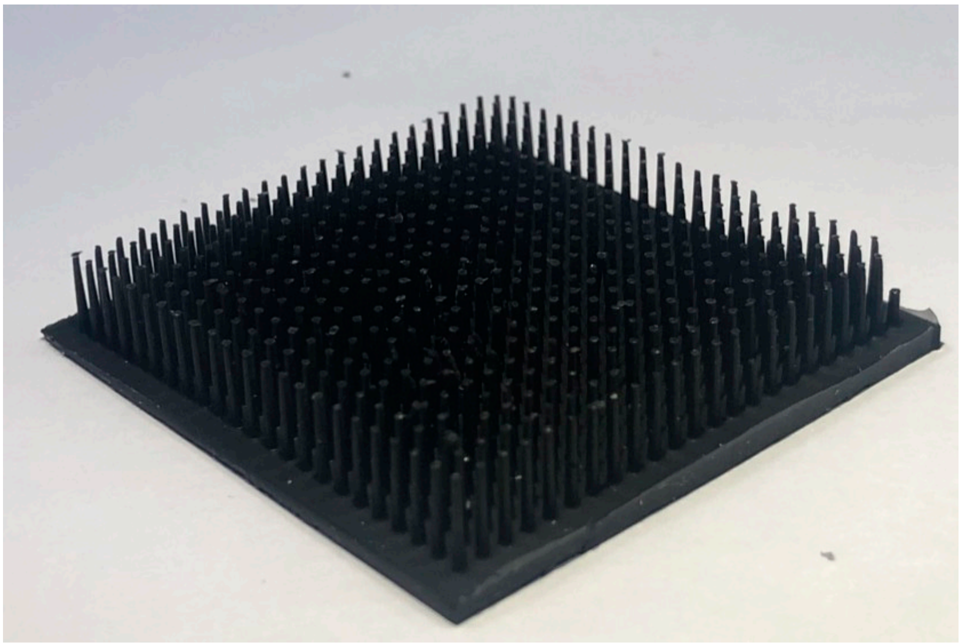

Figure S3. Schematic diagram of the ACMSs sample.

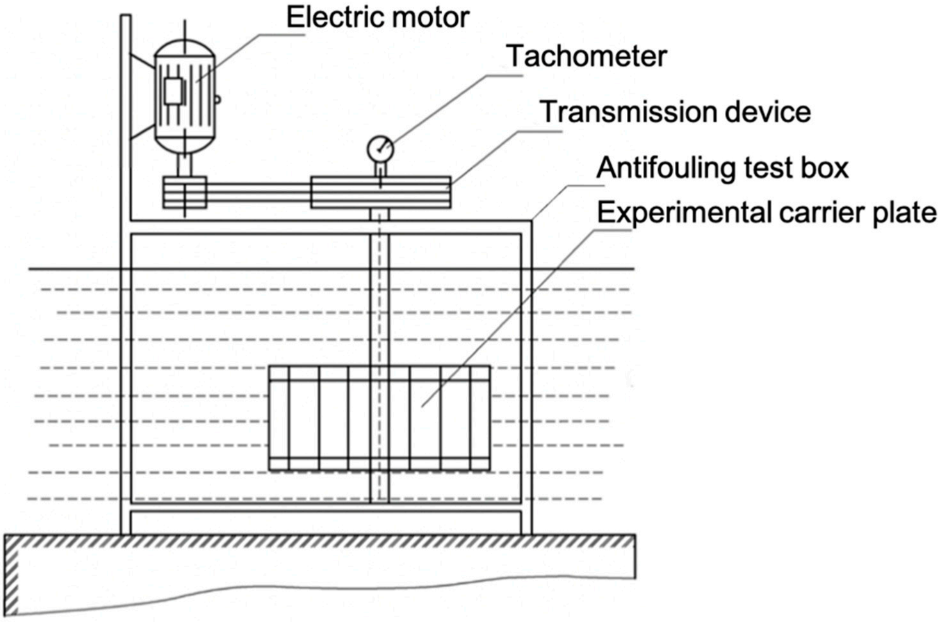

Figure S4. Schematic diagram of the dynamic antifouling rotating scouring device.

It is driven by the electric motor acting on the transmission device to carry out the rotational motion of the experimental carrier plate. Through the rotary motion instead of the linear motion, it can simulate the effect of the ACMSs being scoured by the water

in a single direction during the ship's movement.

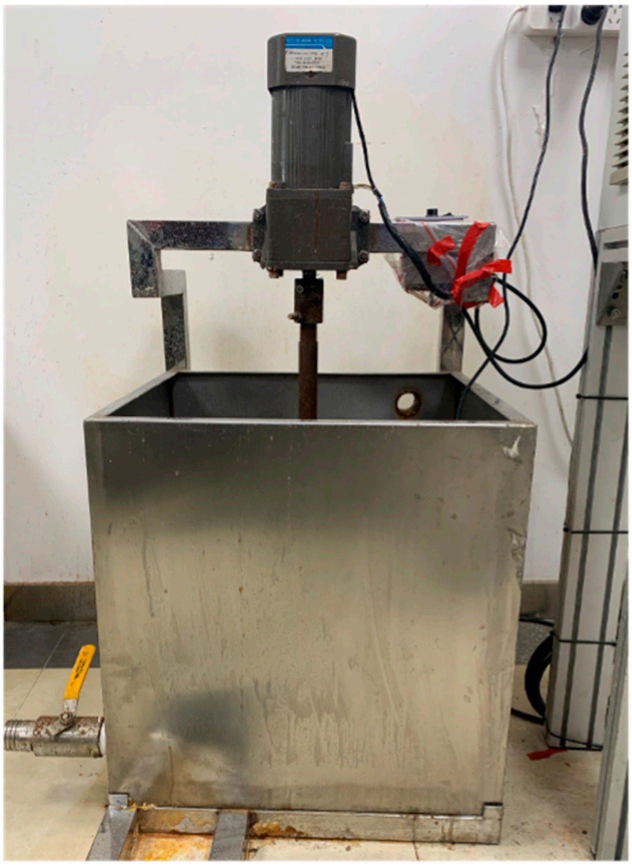

Figure S5. Physical view of dynamic antifouling rotating scouring device.
